# RNA-Based Biomarkers for Diagnostic Discrimination of Ischemic and Hemorrhagic Stroke: A Systematic Review

**DOI:** 10.3390/jcm15041392

**Published:** 2026-02-10

**Authors:** Jan Emmerich, Aditya Chanpura, Frank C. Barone, Alison E. Baird, Tyler M. Lu, Kristian Barlinn, Ben W. M. Illigens, Arturo Tamayo, Hagen B. Huttner, Timo Siepmann

**Affiliations:** 1 Department of Neurology, Medical Faculty and University Hospital Carl Gustav Carus, TUD Dresden University of Technology, 01307 Dresden, Germany; jan.emmerich@ukdd.de (J.E.);; 2 Division of Health Care Sciences, Dresden International University, 01067 Dresden, Germany; 3 Department of Neurology, SUNY Downstate Health Sciences University, Brooklyn, 11203 NY, USA; 4 Molecular and Cellular Biology Program, SUNY Downstate School of Graduate Studies, Brooklyn, 11203 NY, USA; 5 Ronald O. Perelman and Claudia Cohen Center for Reproductive Medicine, Weill Cornell Medicine, New York, 10021 NY, USA; 6 WRHS, BRHA Winnipeg and Brandon Manitoba, Section of Neurology, Department of Medicine, The Max Rady Faculty of Health Sciences, University of Manitoba, Winnipeg, MB R3E 3P5, Canada

**Keywords:** biomarker, diagnostic, intracerebral hemorrhage, ischemic stroke

## Abstract

**Background**: Diagnostic discrimination between ischemic stroke (IS) and hemorrhagic stroke (HS) is required for successful intervention with time-critical acute treatments. The available data on blood-based RNA biomarkers and discrimination between IS and HS are limited. This systematic review aimed to examine and summarize the existing literature on potentially useful blood-based RNA biomarkers that may aid in preclinical acute diagnosis. **Methods**: We systematically reviewed the literature on the ability of blood-based RNA biomarkers to discriminate between IS and HS according to PRISMA guidelines. We searched PubMed, EMBASE, The Cochrane Library, and The Web of Science for eligible randomized controlled trials, observational studies, and case–control studies published in the English language without time limitation. The risk of bias was evaluated using the Newcastle–Ottawa Scale. **Results**: We included eight studies with a total of 728 patients (436 with IS and 292 with HS) in our review. The study quality was good in five and fair in three investigations. No meta-analysis was performed due to high heterogeneity in methods and study endpoints. Reported biomarkers include miRNA-124-3p, miRNA-16, miRNA-340-5p, lncRNA XIST (X-inactive specific transcript), PFKFB3 mRNA (6-phosphofructo-2-kinase/fructose-2,6-biphosphatase), tRNA derivatives, tRNA fragments, extracellular miRNAs, transcriptome changes, and MCEMP1 gene expression. Assessment techniques varied widely across studies, ranging from RNA sequencing to qPCR, microarray, human transcriptome array, and ELISA. MicroRNA-124-3p, miRNA-340-5p, lncRNA XIST, PFKFB3 mRNA, and MCEMP1 gene expression differed significantly between IS and HS. In one study, principal component analysis and unsupervised learning demonstrated the utility of hierarchical clustering of differentially expressed exons to discriminate between HS and IS. **Conclusions**: This review demonstrates the utility of single RNA-based targets and clusters that may have diagnostic value in distinguishing IS from HS. However, the current body of evidence is limited by considerable methodological heterogeneity between studies. **Registration**: This systematic review was prospectively registered on PROSPERO on 21 April 2023 (CRD42023411203).

## 1. Introduction

Stroke is the second leading cause of death worldwide and the third leading cause of long-term disability and death combined [1]. Timely and accurate identification of acute hemorrhagic and ischemic stroke is of paramount importance. This is because the initiation of reperfusion, neurosurgical, and other treatments to counteract functional deterioration and the loss of 1.9 million neurons per minute is time dependent [1,2]. Getting stroke patients to the right hospital with the right level of expertise is critical to patient outcomes [3]. In addition, precise and accurate patient assignment can make a difference for patients while reducing wasted resources such as personnel, unnecessary transport, and response time [3,4]. Implementation of mobile brain computed tomography (CT) on board emergency vehicles (mobile stroke units) has been associated with rapid and reliable detection of ischemic stroke. Immediate exclusion of intracranial hemorrhage has allowed for higher rates of intravenous thrombolysis [5]. Blood-based protein biomarker testing is expected to be less costly, as it requires less technology and fewer personnel [6]. Previous studies investigating blood-based protein biomarkers to differentiate acute IS from HS have included apolipoprotein C-I, apolipoprotein-III, brain natriuretic peptide, glial fibrillary acid protein, matrix metalloproteinase-9, neuron-specific enolase, C-reactive protein, soluble receptor for advanced glycation end products, and S100B [7,8,9,10,11]. To date, no suitable blood-based protein biomarker has been found to reliably identify or differentiate acute IS from HS [7,8,9,10,11].

The decoding of DNA in 2003 led to rapid technical development in the field of genetic diagnostics, with RNA blood-based biomarkers coming to the forefront [12,13]. RNA blood-based biomarkers offer several advantages over protein-based biomarkers, such as ease of detection at very low levels, as sufficient detection can be achieved with less than ten copies of the target of interest [14]. In addition, RNA biomarkers not only provide a dynamic view of the disease under investigation, but expression levels are also more responsive to change than protein biomarkers, as proteins are the result of transcription and translation [15]. Thus, with the advent of new genetic diagnostic techniques, researchers may have begun to investigate the potential utility of RNA blood-based biomarkers for differentiating IS from HS. Accordingly, this study aimed to provide a comprehensive review of the diagnostic discrimination between IS and HS blood RNA, with a particular focus on their potential use as diagnostic markers.

## 2. Materials and Methods

### 2.1. Protocol/Inclusion, Exclusion Criteria

The review was conducted and presented according to the recommendations of the PRISMA (Preferred Reporting Items for Systematic Reviews and Meta-analyses) statement (Tables S1 and S2), and the protocol was registered in Prospero (CRD42023411203) [16,17]. Ethical approval was not required for the present study due to the nature of the data, which was extracted from the results of previous studies.

Inclusion criteria: For this systematic review, we implemented the PICOS question-investigation approach and included patients aged ≥18 years. The intervention group was defined as patients with acute IS. The control group consisted of patients suffering from HS, and the expression levels of RNA-based biomarkers between IS and HS patients determined the outcome. Both targeted and untargeted RNA studies were included to represent the current state of the existing literature. There were no restrictions regarding RNA types (e.g., mRNA, miRNA, tRNA, piwiRNA, snRNA, circRNA, etc.), with blood samples drawn within 72 h of symptom onset.

Exclusion criteria: Studies were excluded if they lacked a control group consisting of HS patients or if they were not published in English.

### 2.2. Search Strategy

Literature searches were conducted by two independent investigators (JR, TL) using EMBASE, The Web of Science, The Cochrane Library, and PubMed publications up to 15 December 2025. The search term combinations used were “stroke [MESH]”, “ischemic stroke”, “intracerebral hemorrhage”, “intracerebral hemorrhage”, “hemorrhagic stroke”, “RNA”, “micro RNA”, “mRNA”, “piRNA”, “piwiRNA”, “snRNA”, “miRNA”, “transcriptome”, with the Boolean operators “AND” and “OR”. The Boolean operator “NOT” was used for the terms “mice”, “mouse”, “rat”, “rats”, and “animal”. Reference lists of identified articles were screened for additional sources. In addition, relevant meta-analyses and systematic reviews were manually screened to ensure a comprehensive review of the literature. The complete search strategy can be found in Table S3. A third reviewer (AC) was involved in case of disagreement between the reviewers, and Zotero^®^ version 6.0.36 was used to screen the search results obtained and to remove duplicates [18].

### 2.3. Data Extraction

The following data were extracted and entered into an Excel spreadsheet (Microsoft, Redmond, WA, USA): (1) study characteristics (type of study, number of patients with IS and HS); (2) country of study; (3) baseline characteristics of participants (age, sex); (4) risk factors (hypertension, diabetes mellitus, etc.); (5) time of blood collection; (6) biomarker measurement method(s); and (7) expression levels of blood-based RNA biomarkers. If data could not be obtained directly by searching the articles for text, tables, and/or supplemental materials, data were extracted using WebPlotDigitizer 4.7.0 [19].

### 2.4. Methodological Quality Assessment—Risk of Bias

All studies were assessed for scientific quality using the Newcastle–Ottawa Scale (NOS). Studies were assessed in the categories of “selection, comparability, and exposure/outcome”, and points were assigned based on study quality [20]. A maximum of nine points could be achieved for the highest possible quality score. As previously described, more than seven points were considered “good quality”, two to six points rated were considered “fair quality”, and one point or less was considered “poor quality” [21].

### 2.5. Synthesis of Results

The heterogeneity in the biomarkers under investigation and the use of disparate detection methods posed significant challenges to the primary objective of conducting a synthetic quantitative analysis. Specifically, the studies examined a broad spectrum of biomarkers, including miR-124-3p, miR-16, Transcriptome, MCEMP1, tRNA-derived fragments, miRNA-340-5p, PFKFB3-mRNA, lncRNA XIST, and tRNA fragments, each with distinct biological roles and varying analytical sensitivities. Additionally, the assays used to quantify these biomarkers varied considerably, with methodologies such as PCR, microarrays, RNA sequencing, HTA, and ELISA being employed across studies. Additionally, the time to blood draw differed widely. These differences in both the target biomarkers and the experimental protocols created substantial heterogeneity in the data, preventing the standardization necessary for a meaningful meta-analysis. Given these issues, a meta-analysis was not feasible.

## 3. Results

### 3.1. Literature Search Results

**Figure 1:**
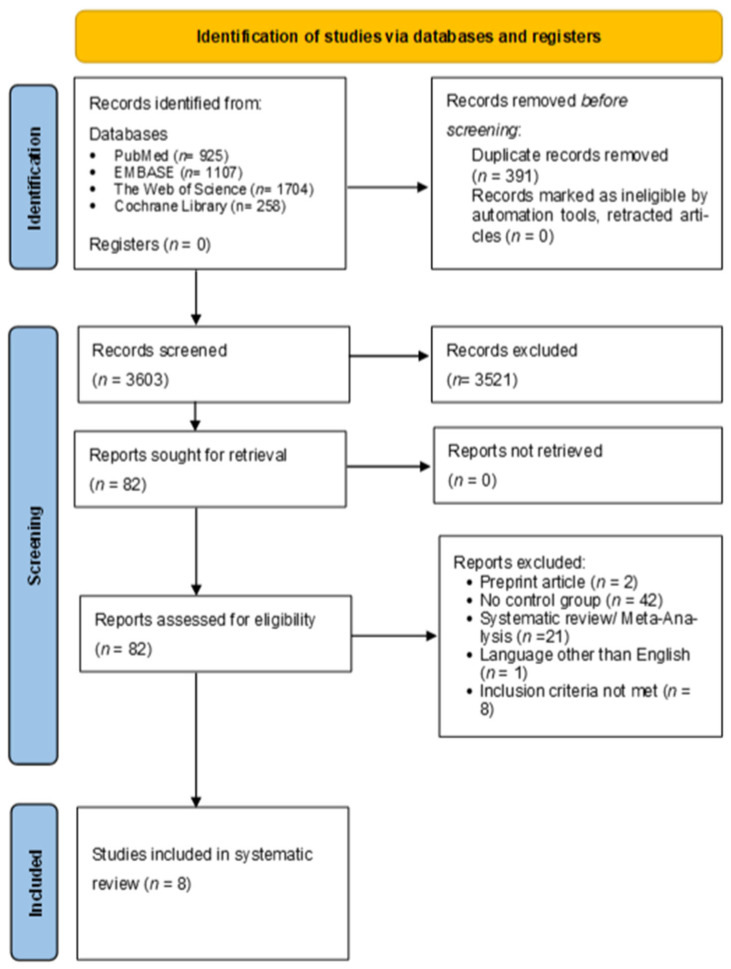
Prism flow chart.

The database search identified a total of 3994 articles from major databases, including PubMed (*n* = 925), EMBASE (*n* = 1107), The Cochrane Library (*n* = 258), and The Web of Science (*n* = 1704). After removing 391 duplicates, 3603 publications were screened based on title and abstract. 3521 articles were excluded because they did not meet the inclusion criteria. After the full-text articles were assessed for eligibility, an additional 74 publications were excluded (reviews [*n* = 21], language other than English [*n* = 1], preprint article [*n* = 2], lack of control group (*n* = 42), and inclusion criteria not met [*n* = 8]). Finally, eight articles were included in this systematic review [22,23,24,25,26,27,28,29]. The PRISMA flowchart is shown in Figure 1.

### 3.2. Study Characteristics

Our analysis includes eight studies with 436 patients with IS and 292 with HS [22,23,24,25,26,27,28,29]. The included studies were conducted between 2014 and 2025 in the USA (3/8), followed by China (1/8), Japan (1/8), Canada (1/8), Egypt (1/8), and the Netherlands (1/8). Two studies looked at the transcriptome, one at tRNA derivatives, one at tRNA-derived fragments, one at miRNA 124-3p and miRNA-16, one at mast cell-expressed membrane protein-1 (MCEMP1) expression, one at extracellular miRNAs, and one at miRNA-340-5p, lncRNA XIST, and PFKFB3 mRNA. The measurement methods used were enzyme-linked immunosorbent assay (ELISA), quantitative polymerase chain reaction (qPCR), RNA sequencing (RNAseq), microarray, and human transcriptome array (HTA) (Table 1a,b).

### 3.3. Quality Assessment

Overall, the assessment of the NOS revealed good quality studies with an average score of 6.63 (standard deviation, SD = 1.32) points. Good quality was found in five of the seven studies included, moderate quality in three, and no studies were found to be of poor quality. Details are shown in Table 2.

### 3.4. Transcriptome Studies

In a recent prospective case–control study of 12 IS and 4 HS patients, the entire transcriptome was analyzed by RNAseq, revealing 412 differentially alternatively spliced (DAS) genes between IS (cardioembolic, CEI; large vessel, LV; lacunar, LAC), HS, and matched controls. Applying a fold change (FC) between HS and IS of >2 for upregulation and of <0.5 for downregulation when examining genes revealed four upregulated DAS and 43 downregulated DAS with a false discovery rate (FDR) of *p* < 0.05 [22]. (Table S4) Of these, 26 DAS were detected between HS and LAC (downregulated: *n* = 24; upregulated: *n* = 2), 24 between HS and LV (downregulated: *n* = 22; upregulated: *n* = 2), and 13 between HS and CEI (downregulated: *n* = 13). Using a cut-off of 2-FC and including all stroke etiologies, no DAS between HS and IS were detected. However, “FAM118A”, “FCER1A”, and “HDC” were differentially spliced among all stroke types and HS when applying a FC < 0.5. Investigated exons showed differential expression (upregulation: *n* = 4; downregulation: *n* = 26) when comparing HS and IS regardless of etiology and applying thresholds mentioned above (FC > 2, FC < 0.5). (Table S5) Of those, HS vs. CEI revealed 1 up- and 15 downregulations, HS vs. LV revealed 3 up- and 15 downregulations, and HS vs. LAC revealed 1 up- and 11 downregulations. Differential exon usage was found in chr1.46467098-46468407 > MAST2 and chr2.88336462-88336570 > KRCC1 when comparing HS and IS regardless of etiology (FC of <0.5; FDR *p* < 0.05). In contrast, another prospective case–control study utilized an HTA to investigate the transcriptomes of 33 HS and 33 IS patients, revealing 256 differentially expressed transcripts (DET) between IS and HS (FDR-corrected by *p* < 0.005) [23]. Of those, three transcripts were significantly upregulated and 14 downregulated, applying a FC threshold of 2 and −2. (Table S6) HS patients showed significantly more DET from T-cell receptors (TCR), (HS: 7% vs. IS: 0%) and protein-coding genes (HS: 52% vs. IS: 38%), but less DET in non-coding RNA (ncRNA, HS: 3% vs. IS: 13%) as well as antisense DET (HS: 0.8–1% vs. IS: 5%). Within the first 24 h, HS had 55 DET from TCR genes, compared to IS patients who showed no DET for TCR. (Table S7) Early discrimination (<24 h) between HS and IS transcriptomes from the control transcriptome was possible based on principal component analysis, which revealed 311 DET in IS and 2667 DET in HS. Blood samples collected within 24 h yielded a total of 2667 DET in HS and 311 DET in IS, allowing differentiation using the controls as a reference. This was also evident when considering different time points (T) (e.g., T1 < 24 h, T2 = 24–48 h, T3 > 48 h). Summarizing all time points, 4537 transcripts of 3259 genes were differentially expressed in HS compared to 1136 transcripts of 1016 genes in IS. The authors also applied pathway enrichment analysis, revealing a predicted activation of IL17A, CSF3, IL1, OSM, TGFA, and HGF in HS but not IS. In addition, it demonstrated a predicted inhibition of IFNB1, FYN, MYC, VHL, and E2F1 in HS but not in IS. SEPT5 was found to be differentially expressed in both studies [22,23]. However, one study found a downregulation of SEPT5 (FC: −1.33) between 24 and 48 h in IS patients compared to controls, while the other study showed an increased expression in patients with large vessel occlusion stroke compared to HS with a mean blood withdrawal time of 47.4 h [22,23].

Overall, studies investigating transcriptomes have shown promise, as they address multiple biomarkers and depict different pathways, potentially providing a more comprehensive ‘fingerprint’ of the disease under investigation. However, the existing data are currently limited, and further research is needed.

### 3.5. Extracellular Vesicles Containing miRNAs

Extracellular vesicles containing RNAs are usually transported within vesicles or in association with RNA-binding proteins or lipoproteins and are thus of significant importance in influencing gene regulation [30,31]. One case–control study found 214, 207, and 240 differentially expressed miRNAs in patients with HS (*n* = 19), subarachnoid hemorrhage (SAH, *n* = 17), and IS (*n* = 21), respectively [24]. The 25 miRNAs with the smallest adjusted *p*-values and the largest FC differences between stroke types were included in the analyses and showed the best discriminative ability for SAH. (Table S8) However, using the 21 most promising miRNAs to discriminate between HS and IS resulted in an area under the curve (AUC) of 0.824 ± 0.001 with an accuracy of 0.811 ± 0.004. For the discrimination between IS and SAH, the AUC was 0.89 ± 0.028 with an accuracy of 0.97 ± 0.065. The best discrimination between SAH and HS patients was observed using the 24 best-matching miRNAs with an AUC of 0.98 ± 0.044 and an accuracy of 0.94 ± 0.086.23 (Table S8). Therefore, it is hypothesized that a single biomarker may not provide sufficient diagnostic accuracy for distinguishing between IS and HS, and biomarker panels could offer a more reliable approach.

### 3.6. Circulating miRNAs/mRNAs/mRNA in Blood and Plasma

In a prospective case–control study of 74 IS and 19 hemorrhagic patients, qPCR detected significantly higher miR-124-3p plasma levels in HS patients compared to IS patients and healthy controls (median plasma levels: 3.8 × 10^5^ vs. 1.9 × 10^5^; 1.5 × 10^5^ copies/mL plasma, respectively) [26]. The calculated AUC for miR-124-3p was 0.70 (95% confidence interval, CI: 0.59–0.79). Using a cutoff of >3 × 10^5^ copies/mL plasma, the optimal sensitivity and specificity were 68.4% and 71.2%, respectively. The highest miR-124-3p levels were measured in HS patients within six hours after symptom onset, while in IS patients the highest levels were found between 6 and 24 h after stroke onset. The biomarker miR-16 was significantly increased by 24% in IS patients compared to HS and healthy controls (1.6 × 10^9^ vs. 1.3 × 10^9^; 1.2 × 10^9^ copies/mL plasma). However, miRNA-16 levels measured within 24 h but 6 h after symptom onset showed a significant difference between IS and HS patients (1.6 × 10^9^ vs. 0.8 × 10^9^ copies/mL, *p* = 0.0061). The reported AUC was 0.66 (95%, CI: 0.55–0.76), and optimal sensitivity and specificity were 94.7% and 35.1%, respectively, using a cutoff of ≤2 × 10^9^ copies/mL plasma. The odds ratio for differentiating HS from IS was 5.37 (>3.0 × 10^5^ copies/mL plasma) for miR-124-3p in plasma and 9.75 (≤2.0 × 10^9^ copies/mL plasma) for miR-16.

Another study investigated the expression levels of microRNA-340-5P in whole blood and revealed a significant decrease in levels in patients with IS and HS compared to healthy controls [0.767, SD: 0.052; 0.423, SD: 0.054] [28]. The lowest expression levels of microRNA-340-5P were found in patients with HS, which differed significantly from patients suffering from IS. Interestingly, both groups, IS as well as HS, comprised the same number of participants (*n* = 40) and were comparable in terms of age and gender. However, a greater proportion of patients with HS also suffered from diabetes mellitus, while a higher percentage of patients with IS suffered from ischaemic heart disease. The discriminatory value of the microRNA-340-5P was reported with an optimal cut-off of 0.63 and an AUC of 0.979 [sensitivity: 97.5%, specificity: 92.5%]. Additionally, the aforementioned study examined the long-coding RNA Xist, which plays a pivotal role in X chromosome inactivation [32]. The highest expression levels were found in patients suffering from HS compared to IS patients, which were found to be significant [3.632, SD: 0.511 vs. 1.587, SD: 0.092]. An optimal cut-off for discrimination between the two entities of 2.02 was reported [AUC: 0.99, sensitivity 95%, specificity 95%]. Another biomarker investigated was the PFKFB3 mRNA, which is ubiquitously expressed in human tissues, with the highest concentration in muscle, fat, kidney, lung, and brain. PFKFB3 mediates glucose metabolism as well as the prevention of apoptosis [33]. Furthermore, PFKFB3 overexpression was revealed in activated inflammatory cells and numerous tumor cells [34]. An observational study found that PFKFB3 mRNA, measured within 24 h of symptom onset, was significantly higher in patients suffering HS compared to IS patients [3.028, SD: 0.372 vs. 1.554, SD: 0.376]. The optimal cut-off was reported to be 2.21, yielding an AUC of 0.98 with a sensitivity of 95% and a specificity of 92.5% [28]. In summary, both mentioned miRNAs and mRNAs appear to be advantageous for distinguishing between IS and HS, given their observed high sensitivity and specificity. However, factors such as cardiovascular diseases, including diabetes mellitus, should be investigated for their influence on expression levels.

### 3.7. tRNA-Derivates/tRNA-Derived Fragments

In a prospective case–control study, tRNA derivatives were tested by ELISA using anti-m1A antibodies in 75 IS and 66 HS patients [25]. The baseline tRNA derivative expression levels in plasma were 232 ng/mL (standard error, SE: 33.1 ng/mL) in IS patients and 212 ng/mL (SE: 23.4 ng/mL) in HS patients, but no difference was found. Nevertheless, tRNA derivatives were significantly higher in both IS and HS patients than in healthy controls at baseline, day 1, day 7, and day 30. The highest values were obtained on day 2 in IS patients [242.3 ng/mL (SE: 31.6)] and on admission in HS patients [212 ng/mL (SE: 23.4)].

Another study investigated tRNA-derived fragments (tRF), aiming to confirm the results of a pilot study that showed discrimination between IS and HS [29]. To enhance the potential clinical applicability of tRF-based biomarkers, specifically analyzed cellular vesicle-depleted plasma. The sum of fragments derived from tRNAs Arg-TCG, Gly-CCC, Leu-CAG, Leu-TAA, Ser-ACT, Ser-GCT, Thr-CGT, Tyr-GTA, and Val-CAC as potential biomarkers was reported. Assuming that the most abundant tRNA-derived fragments contribute most to diagnostic performance, for each parent tRNA, the fragment with the highest read count in the original RNA-sequencing data as the RT-qPCR target was selected. An increase in plasma tRF-TyrGTA1–19 levels in IS patients showed a trend toward higher expression compared with ICH patients (*p* = 0.1041). Additional trends toward differential, although not statistically significant, expression were observed for tRF-ArgTCG53–67 (IS vs. ICH: *p* = 0.0705). A “common tRF model” was constructed, consisting of a combination of three isodecoders (distinct tRNA genes sharing the same anticodon): tRNA-TyrGTA, tRNA-ThrCGT, and tRNA-ValCAC. The highest diagnostic accuracy for discriminating ICH patients from IS patients and stroke mimics was observed for TyrGTA9–29 (AUC: 0.485; 95% CI: 0.359–0.611) [29].

Both tRNA derivates and tRNA fragments appear to have limited potential for distinguishing between IS and HS, possibly due to influences such as caloric intake, with current data remaining insufficient to draw definitive conclusions.

### 3.8. Mast Cell-Expressed Membrane Protein1 Gene Expression (MCEMP1)

Based on the “INTERSTROKE” study cohort, including stroke patients from 22 countries, the first investigation (discovery phase) using microarrays, MCEMP1, exhibited the greatest upregulation following stroke [35,36]. A 2.1-fold increase in MCEMP1 (95% CI, 1.4–3.1; *p* = 3.9 × 10^−4^) was observed in HS compared with IS, with an AUC of 0.75 (95% CI, 0.65–0.85). The highest levels were obtained within 24 h of HS or IS [27]. A discovery phase subset of 57 HS patients and 19 IS patients showed a statistical difference in MCEMP1 expression levels using qPCR. For further comparison, a smaller validation cohort comprising 28 stroke patients and 34 controls recruited from the hospital or within the community without a history of stroke was assessed. This validation group was independent of the Interstroke cohort, which included 24 IS patients and four HS patients, and showed a 4.4 FC in HS patients compared to IS patients, although no statistical significance was found [27]. In control patients, no association was found between MCEMP1 and stroke risk factors (age, sex, ethnicity, body mass index, migraine, hyperlipidemia, atrial fibrillation, diabetes mellitus, and smoking status), except for a moderate association for hypertension (FC: 1.2; 95% CI: 1.1–1.4).

Given the obtained results, it is crucial to establish standardized protocols for the measurement, threshold determination, and interpretation of expression levels. This will enhance comparability across studies and enable more accurate conclusions to be drawn from the data.

## 4. Discussion

Our systematic review reveals that, although research to identify RNA-based biomarkers for differentiating between IS and HS has increased in recent years, the substantial heterogeneity in the selected diagnostic targets, as well as the scientific methods applied in the included studies, make it impractical to synthesize conclusive data on the diagnostic accuracy of specific markers [22,23,24,25,26,27,28,29]. As these studies were exploratory, possible influences of premorbidities were not taken into account. For example, it has been shown that TCR genes are overexpressed not only in HS but also in autoimmune, neurological, and infectious diseases, and may have influenced the results obtained [23,37,38,39]. A high activation of TCR genes is to be expected after HS, as a strong immune reaction can be observed [19]. However, no DET of TCR were detected in the condition of IS, although the activation of an inflammatory cascade after IS has been reported [40,41,42]. Therefore, in-depth studies with larger sample sizes and controlling for pre-morbidities are needed to determine potential clinical benefits.

A promising approach to studying not only pathway-related cell-to-cell interactions but also RNA-based biomarkers is the examination of extracellular vesicles (EVs), which are cell-derived vesicles surrounded by a lipid membrane containing molecules such as nucleic acids, reactive lipids, RNAs, and proteins [43,44]. EVs contain high concentrations of RNAs (e.g., miRNAs, mRNAs, etc.) that can be easily isolated, although these are only a fraction of the circulating RNAs that can be detected in the bloodstream [45]. Since no single EV-related miRNA showed a significant difference in upregulation between IS and HS, it might be appropriate to use miRNAs with the smallest adjusted *p*-values and the largest FC to build a prediction model [24]. One case–control study revealed that the combination of the most promising miRNAs showed a high accuracy in discrimination between SAH and IS, as well as HS [24]. Therefore, combining multiple differentially expressed targets may be a promising approach to increase diagnostic accuracy. This seems reasonable, as a single miRNA usually has the potential to influence several genes and therefore may have low specificity for individual diseases [46,47].

However, in another study, significantly higher miRNA-124-3p copies in IS patients compared to HS patients within six hours of symptom onset were observed [26]. Interestingly, miRNA-124a is brain-cell-specific and therefore appears to be a promising biomarker [26,48]. However, it should be noted that no adjustments for underlying neurological disorders were made. Mostly miRNA-124-3p levels were shown to be decreased in patients suffering from Alzheimer’s, Parkinson’s, multiple sclerosis, and amyotrophic lateral sclerosis, which should be taken into account [49,50]. Furthermore, miRNA-124-3p appears to counteract the progression of post-stroke inflammation by regulating neuroinflammation, neuronal excitability, and neurodifferentiation by playing a key role in the miR-124/DAPK1 signaling pathway [51]. Expression of miRNA-16 differed significantly between IS and HS patients between 6 h and 24 h after symptom onset. However, miRNA-16 seems to be less stroke-specific, as elevated levels have been found in cancer and rheumatoid arthritis, and it plays a key role in conditions like systemic lupus erythematosus, inflammatory bowel disease, type 1 diabetes, and multiple sclerosis [52,53]. A limitation of this pilot study is its reliance on a previously conducted rodent study, which indicated a higher prevalence in the brain, thus introducing the potential for bias; therefore, not only neurological, but also cardiovascular risk factors, as well as disorders, should be taken into consideration in future studies.

Another promising miRNA could be miRNA-340-5p, which has been identified as a specific target of regulation in the context of neural protection across a range of experimental models [54,55]. Furthermore, it has been observed to interact with the long non-coding RNA XIST, which has been detected in patients with atherosclerosis and myocardial infarction, being considered a high-risk factor for strokes [56,57,58,59,60]. However, it is important to note that the patients were not evenly distributed with respect to risk factors for ischemic heart disease and diabetes mellitus, which may have influenced the observed results, particularly given that miRNA-340-5p was found to be overexpressed in a mouse model of diabetes and cardiac dysfunction [61]. Further research is required to confirm these observations in a standardized fashion, elucidating the potential impact of diabetes mellitus and ischaemic heart disease, and to incorporate these as a covariate in subsequent studies. It is important to consider the potential interaction between the miRNA-340-5P and the lncRNA XIST, as this could potentially influence the measured effect of each marker. This is particularly important, as studies have shown that microRNA-340 is responsive to insulin and glucose stimuli in cultured lymphocytes, suggesting a potentially critical role in the changes in gene expression induced by hyperinsulinemia [62]. The observed association between diabetes mellitus, cardiovascular diseases, and stroke with the expression of miRNA-340-5P and lncRNA XIST necessitates further investigation. The diagnostic potential of these biomarkers for acute diagnostic purposes should be explored, given that stroke patients often suffer from concomitant diabetes and cardiovascular conditions.

PFKF3B (6-phosphofructo-2-kinase/fructose-2,6-biphosphatase) is an enzyme that mediates glucose metabolism and may be elevated in several diseases where increased glucose metabolism is required or is present, such as cancer or inflammation, e.g., rheumatoid arthritis, atherosclerosis, or cardiovascular diseases [33,63,64,65,66,67]. In all groups studied, patients exhibited varying degrees of diabetes mellitus and ischaemic heart disease, which may have resulted in a distortion of the PFKFB3 mRNA expression values. Further large-scale studies are required to ascertain whether groups matched for these and other factors can withstand the results of the observational study.

Useful RNA-based biomarkers must not only be highly sensitive and specific for the disease of interest but also provide procedurally stable results. Interlaboratory variability in measurement results due to different protocols or measurement techniques can be challenging. For instance, transcriptome studies carried out using microarrays offer the advantage that several thousand targets can be examined [68]. However, precision can be low, and results can either be false negative or false positive [68]. Whenever possible, obtained results should be validated with qPCR or RNAseq due to higher sensitivity and specificity [68,69]. Two of the studies included in our review dealt with microarray, while just one of them validated their results by applying a qPCR [23,26,27]. It has been shown that initially significant results obtained with the microarray were no longer statistically significant when validated with qPCR, although it should be noted that only four patients were studied in the validation phase [27]. Since the sample size of transcriptomics studies is critical to the number of transcripts measured, the included studies are limited in their power. In addition, RNAs identified without validation may be false positives even with FDR correction, so qPCR remains the gold standard for gene expression analysis [69,70,71].

Another promising approach is RNAseq, which provides a higher measurement precision of transcript levels compared to microarrays [72]. The quality of the RNAseq reads is assessed by the “PHRED” score, which was 37 in one of the studies included in this manuscript, indicating an error rate of less than 0.1% [23,24,73]. Regardless of the measurement methods used, it is important not only to present the results as FC but also to incorporate statistical testing, as high FC does not automatically indicate a significant result [27], e.g., a FC of 4.4 between MCEMP1 levels in IS and HS patients was observed, albeit no statistical significance was detected [27]. This can be due to several reasons, including overlapping confidence intervals, small sample size, and overcorrection of statistical testing [74]. Furthermore, raw FCs are unreliable when not considering the uncertainty of gene expression measurements under the two conditions being compared [74]. FCs in genes with a lower read count are less reliable than in high read count genes [75]. Additionally, the use of FC in combination with FDR and/or various corrections is required in multiple testing, e.g., the Bonferroni correction can provide greater certainty in interpretation [75,76,77].

In addition to the technical challenges of correctly identifying differentially expressed RNA-based targets, it should be noted that RNA is degraded by internal RNAse in the blood, requiring rapid and appropriate processing [78]. It should be considered that shorter RNAs, such as miRNAs, can be influenced by pre-existing conditions such as hypertension, gender, or age differences [79,80,81]. Therefore, pre-existing conditions should be accounted for, and comparable baseline conditions should be present.

Caloric intake may also have had a substantial impact on tRNA derivative expression [25]. In addition, tRNA derivatives were measured using an indirect sandwich ELISA, which is commonly used to measure protein concentrations [25]. Overall, tRNA subgroups and the influence of caloric intake should be investigated in future studies using RNA-specific assay techniques [82].

In summary, miRNA-124-3p, miRNA-340-5p, PFKFB3 mRNA, lncRNA XIST, as well as MCEMP1, could be potential single RNA-based biomarkers to differentiate IS from HS [26,27,28]. Nevertheless, cluster identification could be a promising approach to studying the composition of the entire transcriptome, since individual miRNAs often encode multiple genes and, therefore, specificity remains low [46,82,83]. Principal component analysis and unsupervised learning could be an appropriate approach to create the fingerprint of subtypes of stroke in the future [22,84,85,86]. However, these conclusions derive from a highly heterogeneous body of evidence, highlighting an urgent need for well-standardized research on the potential utility of RNA-based biomarkers to discriminate between IS and HS. The substantial methodological heterogeneity of the included studies and the exploratory nature of the studies limit our review. Importantly, no universally valid thresholds for the magnitude of expression levels have been established to date, so the interpretation of research on this topic must be considered in the context of the individual study, further limiting the external validity of the results presented.

## 5. Conclusions

RNA-based biomarkers have the potential to aid in the diagnostic differentiation of IS and HS; however, their clinical application is currently limited by significant methodological heterogeneity and the lack of clear, universally accepted threshold values. Given the multifaceted nature of stroke pathophysiology, it is unlikely that single biomarkers alone will provide sufficient diagnostic precision. To enhance their clinical utility, future research should prioritize the development of standardized biomarker panels, accompanied by pre-defined thresholds. Such an approach would facilitate more reliable identification of relevant transcriptional patterns and advance the translation of stroke biomarkers into clinical practice, particularly within neurology and neurosurgery.

## Figures and Tables

**Table 1:** (**a**) Characteristics of the included studies in the systematic review. N = Number of patients, SD = Standard Deviation, SE = Standard Error, IQR = Inter Quartile Range, n.a. = Not Applicable, CEI = Cardio Embolic Infarction, LV = Large Vessel Occlusive Stroke, LAC = Lacunar Stroke, IS = Ischemic Stroke, SAH = Subarachnoid Hemorrhage, HS = Hemorrhagic Stroke, SM = Stroke mimic, h = hours, HTN = Hypertension, HLP = Hyperlipoproteinemia, D.m. = Diabetes mellitus. (**b**) Results of the Included Studies in the Systematic Review. AUC = Area Under Curve n.a. = Not Applicable, IS = Ischemic Stroke, SAH = Subarachnoid Hemorrhage, HS = Hemorrhagic Stroke, SM = Stroke Mimics, ELISA = Enzyme-linked Immunosorbent Assay, qPCR = quantitative Polymerase Chain Reaction, DAS = Differential Altered Spliced Genes, DET = Differentially Expressed Transcripts, TCR = T-Cell Receptor.

**(a)**									
**Author**	**Year**	**Country**	**IS (N)**	**IS, Age** **[Mean (SD)]**	**HS (N)**	**HS, Age [Mean (SD)]**	**Blood Collecting Time in h, Mean (SD), Median [IQR]**	**Risk Factors**	
								**Ischemic Stroke**	**Ischemic Stroke**
Leung et al. [26]	2014	China	74	n.a.	19	n.a.	7.5 [9.6]	n.a.	n.a.
Dykstra-Aiello et al. [22]	2016	USA	CEI: 4 LV: 4 LAC: 4	CEI: 62.3 (9.6) LV: 61.0 (8.2) LAC: 58.9 (9.0)	4	60.1 (2.3)	CEI: 33.7 (18.9) LV: 47.4 (47.8) LAC: 34.6 (23.7) ICH: 29.4 (15.5)	HT: CEI: 4/4; LV: 4/4; LAC: 2/4 D.m.: CEI: 2/4; LV: 2/4; LAC: 0/4 HLP: CEI: 3/4; LV: 2/4; LAC: 2/4	HTN: 3/4 D.m.: 4/4 HLP: 4/4
Raman et al. [27]	2016	Canada	Discovery phase (*n* = 104): ≤24 h, *n* = 12 24–48 h, *n* = 31 48–72 h, *n* = 35 72–96 h, *n* = 22 96+ h, *n* = 4 Subset: *n* = 19 Validation phase: *n* = 24	n.a.	Discovery Phase (*n* = 25): ≤24 h, *n* = 6 24–48 h, *n* = 7 48–72h, *n* = 8 72–96 h, *n* = 4 96+ h, *n* = 0 Subset: *n* = 57 Validation phase: *n* = 4	n.a.	Discovery phase: 52.1 (23.7) Subset: n.a. Validation phase: 63.2 (26.5)	n.a.	n.a.
Stamova et al. [23]	2019	USA	33	64.8 (13.0)	33	62 (14.3)	IS: 48.5 (28) HS: 57.3 (30.6)	HTN: 25/33 D.m.: 6/33 HLP: 12/33 Current Smoker: 9/33	HTN: 23/33 D.m.: 6/33 HLP: 6/33 Current Smoker: 7/33
Ishida et al. [25]	2020	Japan	75	72.1 (SE:1.32)	66	64.3 (SE:1.52)	IS: 6.02 (SE:0.85) HS: 5.08 (SE:1.06)	HTN: 42/75 D.m.: 20/75 Prior Stroke: 9/75 Current Smoker: 26/75 BMI > 25: 21/75 Alcohol use: 24/75 Coronary disease: 13/75	HTN: 54/66 D.m.: 16/66 Prior Stroke: 6/66 Smoker: 24/66 BMI > 25: 9/66 Alcohol use: 17/66 Coronary disease: 4/66
Kalani et al. [24]	2020	USA	21	66 (n.a.)	SAH: *n* = 17 HS: *n* = 19	SAH: 58 (n.a.) HS: 65 (n.a.)	<24 h after last seen normal	Smoker: 3/21 HTN: 13/21	SAH: Smoker: n.a. HTN: n.a. HS: Smoker: 3/19 HTN: 15/19
Elhorany et al. [28]	2024	Egypt	40	53.05 (4.91)	40	53.25 (5.79)	<24 h	HTN: 19/40 D.m.: 7/40 Smoker: 10/40 Heart Disease: 13/40	HTN: 23/40 D.m.: 16/40 Smoker: 7/40 Heart Disease: 2/40
Woudenberg et al. [29]	2025	The Netherlands	35	71 (15)	25	71(11)	<6 h	HTN: 22/34 D.m. 7/34 Hyperlipidemia: 18/34 Myocardial infarction: 5/34 Ischemic Stroke/TIA: 10/34 Intracerebral hem.: 2/34	HTN: 10/25 D.m. 2/25 Hyperlipidemia: 11/25 Myocardial infarction: 4/25 Ischemic Stroke/TIA: 7/25 Intracerebral hem.: 1/25
**(b)**									
**Author**	**Target**		**Method**	**Results**					
				**AUC (95% CI)**	**Sensitivity**	**Specificity**	**Upregulation vs. Downregulation**		
Leung et al. [26]	miR-124-3p		qPCR	0.70 (0.59–0.79), cutoff > 3 × 10^5^ copies/mL plasma,	68.4%	71.2%	(0–6 h), *p* = 0.0217		
	miR-16			0.66 (0.55–0.76), cutoff of ≤2 × 10^5^ copies/mL plasma	94.7%	35.1%	(0–6 h), *p* > 0.05; (6–24 h): *p* = 0.0061		
Dykstra-Aiello et al. [22]	Transcriptome		RNA-Seq.	n.a.	n.a.	n.a.	Expression rate > 2-fold IS vs. HS: DAS: none; Exons: none Expression rate < 0.5 fold IS vs. HS: DAS: FAM118A; FCER1A; HDC Exons, FC < 0.5: chr1.46467098-46468407 > MAST2 chr2.88336462-8833 570 > KRCC1		
Raman et al. [27]	MCEMP1		Discovery phase: Microarray	0.75 (0.65–0.85)	n.a.	n.a.	Discovery phase (all times) HS vs. IS: *p* < 0.0005 HS vs. IS: 2.1 fold, *p* = 3.9 × 10^−4^		
			Subset: qPCR	n.a.	n.a.	n.a.	Subset (all times) HS vs. IS: *p* < 0.05		
			Validation phase: qPCR	n.a.	n.a.	n.a.	Validation phase (all times) HS vs. IS: *p* = 0.074 (HS vs. IS: FC > 4.4)		
Stamova et al. [23]	Transcriptome		HTA	n.a.	n.a.	n.a.	FC > 2, *p* < 0.05: EVL-017; RAB27A-002; TBC1D8-011 FC < (−2), *p* < 0.05: ANKH-201; AP2B1-012; APOBEC3G-004; ITGB7-006; LEF1-005; LEF1-009; LMNB1-004; N.A.P1L1-008; PTPN4-004; RHCE-002; RP11-175P13.3-001; RRM1-014; SLC16A10-004; TTC39B-204 T-cell-Receptors: HS: *n* = 55 DET from TCR genes vs. IS *n* = 0 DET for TCR		
Ishida et al. [25]	tRNA- derivates		ELISA	n.a.	n.a.	n.a.	IS vs. HS tRNA derivate levels (day 0): *p* = 0.7277 Infarction volume vs. tRNA derivates: (r = 0.445, *p* = 0.00018). Hematoma volume vs. tRNA derivates: (r = 0.34, *p* = 0.0072).		
Kalani et al. [24]	Extracellular miRNA		RNA-Seq.	IS vs. SAH + HS: 0.752 ± 0.003 Accuracy: 0.816 ± 0.003 IS vs. SAH: 0.89 ± 0.028 Accuracy: 0.97 ± 0.065 SAH vs. HS: 0.98 ± 0.044 Accuracy: 0.94 ± 0.086 SAH vs. IS + HS: 0.927 ± 0.009 Accuracy: 0.97 ± 0.002 HS vs. IS: 0.824 ± 0.001 Accuracy: 0.81 ± 0.004	n.a.	n.a.	n.a.		
Elhorany et al. [28]	miRNA-340-5P		qPCR	0.979 (n.a.), cutoff = 0.63	97.5%	92.5%	HS vs. IS: 0.423 ± 0.054 and 0.767 ± 0.052, *p* < 0.05		
	PFKFB3 mRNA			0.98 (n.a.), cutoff = 2.21	95%	92.5%	HS vs. IS: 3.028 ± 0.372 and 1.554 ± 0.376, *p* < 0.05		
	lncRNA XIST			0.99 (n.a.), cutoff = 2.02	95%	95%	HS vs. IS: 3.632 ± 0.511 and 1.587 ± 0.092, *p* < 0.05		
Woudenberg et al. [29]	t-RNA fragments		qPCR	ROC analysis HS vs. IS + SM ValCAC: 0.412 (0.282–0.542) TyrGTA: 0.485 (0.359–0.611) ThrCGT: 0.484 (0.350–0.619)	n.a.	n.a.	Common-tRF model: IS vs. HS + SM: 0.544 (0.413–0.666) HS vs. IS + SM: 0.371 (0.238–0.504)		
				ROC analysis IS vs. HS + SM ValCAC: 0.560 (0.434–0.686) TyrGTA: 0.574 (0.444–0.705) ThrCGT: 0.552 (0.428–0.0675)					

**Table 2:** Risk of bias assessment, Newcastle–Ottawa Quality Assessment Scale. Denotes the NOS star rating (one star [*] = one point); “-” indicates no star given.

Author	Study Year	Selection 1	Selection 2	Selection 3	Selection 4	Comparability	Exposure Outcome 1	Exposure Outcome 2	Exposure Outcome 3	Total	Study Quality
Leung et al. [26]	2014	*	*	*	-	-	*	*	-	5	2
Dykstra-Aiello et al. [22]	2015	*	*	*	*	**	*	*	-	8	1
Raman et al. [27]	2016	*	*	*	*	-	*	*	-	6	2
Stamova et al. [23]	2019	*	*	*	-	**	*	*	-	7	1
Ishida et al. [25]	2020	*	*	*	*	-	*	*	-	6	2
Kalani et al. [24]	2020	*	*	*	-	-	*	*	-	5	2
Elhorany et al. [28]	2024	*	*	*	*	**	*	*	*	9	1
Woudenberg et al. [29]	2025	*	*	*	*	*	*	*	-	7	1

## Data Availability

No new data were created or analyzed in this study. Data sharing does not apply to this article.

## Supplementary Materials

The following supporting information can be downloaded at: https://www.mdpi.com/article/10.3390/jcm15041392/s1, Table S1: PRISMA 2020 for Abstracts Checklist; Table S2: PRISMA 2020 Checklist; Table S3: Search Strategy; Table S4: Differentially alternative splicing genes in introns (Dykstra-Aiello et al.) [22]; CEI = Cardio Embolic Infarction, LV = Large Vessel infarction, LAC = Lacunar infarction, ICH = Intracranial Hemorrhage, Ave Average, SD = Standard Deviation, FC = Fold Change, (-) = For clarity, the representation of 2 < FC > 0.5 is omitted; Table S5: Differentially alternative splicing genes in exons (Dykstra-Aiello et al.) [22]; CEI = Cardio Embolic Infarction, LV = Large Vessel infarction, LAC = Lacunar infarction, ICH = Intracranial Hemorrhage, Ave = Average, SD = Standard Deviation, FC = Fold Change, (-) = For clarity, the representation of 2 < FC > 0.5 is omitted; Table S6: Differentially expressed genes (Stamova et al.) [23]; FC = Fold Change, ID = Identification; IS = Ischemic Stroke, HS = Hemorrhagic Stroke; Table S7: Differentially expressed transcriptomes from TCR genes comparing HS and controls, extracted from (Stamova et al.) [23], FC = Fold change, TR = T-cell receptor; Table S8: Extracellular miRNAs selected by LASSO analysis with best discriminatory power (Kalani et al.) [24], SAH = Subarachnoid Hemorrhage, IS = Ischemic Stroke, HS = Hemorrhagic Stroke.

## Abbreviations

AUC	Area Under Curve
CE	Cardioembolic stroke
CT	Computer Tomography
DAS	Differentially Alternatively Spliced genes
DET	Differentially Expressed Transcripts
ELISA	Enzyme-Linked Immunosorbent Assay
EV	Extracellular Vesicle
FC	Fold Change
FDR	False Discovery Rate
IS	Ischemic Stroke
HS	Hemorrhagic Stroke
HTA	Human Transcriptome Array
LAC	Lacunar Stroke
lncRNA XIST	Long coding RNA (X-inactive specific transcript)
LV	Large Vessel occlusive stroke
MCEMP1	Mast Cell-Expressed Membrane Protein-1
NOS	Newcastle–Ottawa Scale
PFKFB3	6-phosphofructo-2-kinase/fructose-2,6-biphosphatase
PRISMA	Preferred Reporting Items for Systematic Reviews and Meta-Analyses
qPCR	Quantitative Polymerase Chain Reaction
RNAseq	RNA Sequencing
SAH	Subarachnoid Hemorrhage
SD	Standard Deviation
SE	Standard Error
TCR	T-Cell Receptor
