# Supplementary material for: RNA-Based Biomarkers for Diagnostic Discrimination of Ischemic and Hemorrhagic Stroke: A Systematic Review
Source: J Clin Med. 2026 Feb 10;15(4):1392. doi: 10.3390/jcm15041392 (PMC12942304; doi:10.3390/jcm15041392)
Supplement: Supplementary file 1 [file jcm-15-01392-s001.zip › Table S3-New_A1_Appendix_Literature_Search_Strategy.pdf]

RNA-based biomarkers for diagnostic discrimination of ischemic and hemorrhagic stroke: A systematic review

Search Strategy

MEDLINE:

"stroke"[MeSH Terms] AND ("micrornas"[MeSH Terms] OR "rna, nuclear"[MeSH Terms] OR "rna, small interfering"[MeSH Terms] OR "rna, messenger"[MeSH Terms] OR "Transcriptome"[Mesh]) NOT ("Mice"[Title/Abstract] OR "Mouse"[Title/Abstract] OR "Rat"[Title/Abstract] OR "Rats"[Title/Abstract] OR "Rat"[Title/Abstract])

EMBASE:

Search restricted to the English language, search up to 15/12/2025, Abstracts are screened, limited to studies involving humans, removing MEDLINE Records

(ischemic stroke or ischaemic stroke or intracerebral hemorrhage or intracerebral bleeding or hemorrhagic stroke) and (MicroRNA or micro RNA or snRNA or small interfering RNA or piRNA or piwiRNA or mRNA or RNA or transcriptome) not (mice or mouse or rat or rats).ti or (ischemic stroke or ischaemic stroke or intracerebral hemorrhage or intracerebral bleeding or hemorrhagic stroke) and (MicroRNA or micro RNA or snRNA or small interfering RNA or piRNA or piwiRNA or mRNA or RNA or transcriptome) not (mice or mouse or rat or rats).ab

The Web of Science:

(AB=(("ischemic stroke" or "ischaemic stroke" or "intracerebral hemorrhage" or "intracerebral bleeding" or "hemorrhagic stroke") and (MicroRNA or "micro RNA" or snRNA or "small interfering RNA" or piRNA or piwiRNA or mRNA or RNA or transcriptome) not (“Mice” or “Mouse” or “Rat” or “Rats”))) OR (TI=(("ischemic stroke" or "ischaemic stroke" or "intracerebral hemorrhage" or "intracerebral bleeding" or "hemorrhagic stroke") and (MicroRNA or "micro RNA" or snRNA or "small interfering RNA" or piRNA or piwiRNA or mRNA or RNA or transcriptome) not (“Mice” or “Mouse” or “Rat” or “Rats”)))

Cochrane Library:

Date Run: 15/12/2025 22:43:15

ID	Search	Hits
#1	MeSH descriptor: [MicroRNAs] explode all trees	930
#2	MeSH descriptor: [RNA] explode all trees	24329
#3	MeSH descriptor: [Stroke] explode all trees	93970

#4	MeSH descriptor: [RNA, Small Nuclear] explode all trees	108
#5	MeSH descriptor: [Piwi-Interacting RNA] explode all trees	4
#6	MeSH descriptor: [RNA, Messenger] explode all trees	2382
#7	MeSH descriptor: [Transcriptome] explode all trees	1375
#8	#1 OR #2 OR #4 OR #5 OR #6 OR #7	25245
#9	#3 AND #8	263
