# Supplementary material for: RNA-Based Biomarkers for Diagnostic Discrimination of Ischemic and Hemorrhagic Stroke: A Systematic Review
Source: J Clin Med. 2026 Feb 10;15(4):1392. doi: 10.3390/jcm15041392 (PMC12942304; doi:10.3390/jcm15041392)
Supplement: Supplementary file 1 [file jcm-15-01392-s001.zip › Table S4-New_A2_Table.pdf]

Marker	Stroke entities								Fold Change (FC)		
	CEI		LV		LAC		ICH		CEI vs. ICH	LV vs. ICH	LAC vs. ICH
**Introns, FC> 2**	**Ave**	**SD**	**Ave**	**SD**	**Ave**	**SD**	**Ave**	**SD**			
SEPT5 and GP1BB	3040	1005	4414	2440	3324	1422	2197	863	-	2.009103323	-
kihire	9478	5748	33285	20787	21896	13674	6453	2899	-	5.158066016	3.393150473
NAPSB	3405	1074	3529	2122	10859	8597	5374	1885	-	-	2.020655006
**Introns, FC< 0.5**	**Ave**	**SD**	**Ave**	**SD**	**Ave**	**SD**	**Ave**	**SD**			
APIP	317	80	533	148	687	446	762	173	0.416010499	-	-
CDKN1C	475	258	793	414	636	513	980	677	0.484693878	-	-
**FAM118A**	**1404**	**474**	**3619**	**4483**	**3284**	**2922**	**11737**	**9061**	**0.119621709**	**0.308341143**	**0.279798926**
**FCER1A**	**659**	**362**	**789**	**540**	**660**	**439**	**2643**	**1657**	**0.249337874**	**0.298524404**	**0.249716232**
GBP4 and GBP7 and GBP2	19307	1526	36610	21937	21192	7897	38993	18687	0.495140153	-	-
GZMA	1428	617	1547	795	1271	996	2937	1502	0.486210419	-	0.432754511
**HDC**	**614**	**503**	**239**	**176**	**302**	**216**	**1764**	**1764**	**0.348072562**	**0.135487528**	**0.171201814**
HERC5	1263	504	2804	1990	943	266	2549	1396	0.495488427	-	0.369949
ITGA4	7230	2523	9755	2452	7895	2840	14878	4886	0.485952413	-	-
LACTB	1209	449	1629	357	1270	382	2434	453	0.496713229	-	-
MIAT	5163	1684	8840	2583	4921	1595	16520	8211	0.312530266	-	0.297881356
MS4A6E and MS4A7 and MS4A14	3961	930	5291	2260	3274	1278	7972	1801	0.496864024	-	0.41068740
SYTL2	609	448	625	273	691	291	1350	698	0.451111111	0.462962963	-
C15orf29	2112	548	1868	754	2435	783	4101	1486	-	0.455498659	-
CYBRD1	2790	1092	1984	890	2450	514	4998	1404	-	0.396958784	0.490196078
DCP2	5982	2195	4905	1487	5189	733	10062	1270	-	0.487477639	-
DMXL2	6338	2435	4960	1513	4926	1774	10167	1770	-	0.487852857	0.484508705
EGLN1	8944	3457	6538	1635	7818	2295	13555	3221	-	0.482331243	-
FAM198B	3287	931	2792	702	2275	924	5879	2203	-	0.474910699	0.386970573
GCA	28956	13098	18438	5801	18391	7008	41971	15709	-	0.439303328	0.438183508
HIPK3	10559	4494	7184	1405	9652	1000	16238	3023	-	0.442419017	-
IFNAR1	7051	2187	6421	2004	7847	374	13900	9175	-	0.461942446	-
IPMK	1096	420	728	194	1034	258	2010	791	-	0.362189055	-
IQGAP2	5359	2087	4799	779	5177	1850	9694	642	-	0.495048484	-
JMJD1C	8720	2885	7092	1945	10897	4177	14207	2528	-	0.49919054	-
MAN1A1	4096	1084	3299	734	3791	562	6939	3097	-	0.475428736	-
NAB1	1265	401	1156	347	1379	402	2315	285	-	0.499352052	-
PELI1	9146	3957	5929	1313	8866	1884	12489	2927	-	0.474737769	-
RGS18	6497	3737	4306	897	4240	984	9388	2786	-	0.458670643	0.451640392
SPOPL	4318	1866	3080	902	3825	413	6948	1440	-	0.443293034	-
TCP11L2	3240	757	1773	295	2781	1019	3742	794	-	0.473810796	-
ANXA1	13211	5310	10788	6389	8183	3672	17739	2842	-	-	0.461299961
CAPZA2	5544	1890	4938	2718	4393	952	8937	1790	-	-	0.491551975
CD36	7954	1387	9710	2338	7659	4520	15417	1591	-	-	0.496789259
CLEC7A	16330	7234	14100	5400	10363	3669	24725	4734	-	-	0.419130435

CSGALNACT2	4666	2003	3319	700	3196	477	6621	446	-	-	0.48270654
DDX60L	6912	2530	9242	5198	5270	2022	11091	3992	-	-	0.47516004
DPYD	5956	2685	5701	1090	5237	2686	10758	610	-	-	0.486800521
LPCAT2 and CAPNS2	5868	1937	5455	2276	3913	1196	8651	1594	-	-	0.452317651
LRRK2	24315	8105	20896	9649	17449	5076	36530	10282	-	-	0.477662195
OAS3	4184	1341	9847	9297	3080	1188	7335	3046	-	-	0.419904567
PARP9	8983	2666	12169	8044	6434	921	13178	5295	-	-	0.488237972
TNFSF13B	4531	1179	5254	2916	3331	778	7693	1852	-	-	0.432991031
