# Supplementary material for: RNA-Based Biomarkers for Diagnostic Discrimination of Ischemic and Hemorrhagic Stroke: A Systematic Review
Source: J Clin Med. 2026 Feb 10;15(4):1392. doi: 10.3390/jcm15041392 (PMC12942304; doi:10.3390/jcm15041392)
Supplement: Supplementary file 1 [file jcm-15-01392-s001.zip › Table S5-New_A3_Table.pdf]

**Marker ID**	**Stroke entities**								**Fold Change (FC)**		
	**CEI**		**LV**		**LAC**		**ICH**		**CEI vs. ICH**	**LV vs. ICH**	**LAC vs. ICH**
**Exons, FC> 2**	**Ave**	**SD**	**Ave**	**SD**	**Ave**	**SD**	**Ave**	**SD**			
chr2.179463448-179463831>CCDC141andTTN	20	9	8	5	6	2	9	3	2.22222	-	-
chr1.214836934-214837426>CENPF	16	5	36	5	15	10	13	8	-	2.769231	-
chr2.173420100-173420447>PDK1	2	3	9	4	1	2	4	2	-	2.25	-
chr3.39162488-39162680>TTC21A	11	4	9	4	38	59	17	5	-	-	2.235294
**Exons, FC< 0.5**	**Ave**	**SD**	**Ave**	**SD**	**Ave**	**SD**	**Ave**	**SD**			
chr1.160580214-160580588>SLAMF1	209	100	332	129	371	208	455	139	0.459341	-	-
chr1.161196029-161196394>TOMM40L	27	19	36	14	50	25	67	31	0.402985	-	-
chr1.201780731-201780885>NAV1	5	6	7	5	15	12	17	8	0.294118	0.411765	-
**chr1.46467098-46468407>MAST2**	**5**	**2**	**2**	**3**	**5**	**3**	**14**	**3**	**0.357143**	**0.142857**	**0.357143**
chr1.46805848-46806591>NSUN4andFAAH	27	19	33	9	46	25	62	10	0.435484	-	-
chr1.78207302-78207433>USP33	76	46	85	46	63	21	170	15	0.447059	-	0.370588
chr2.110584278-110584424>RGPD5	24	9	30	14	34	10	66	6	0.363636	0.454545	-
chr2.111302237-111302383>RGPD6	24	9	30	14	34	10	65	7	0.369231	0.461538	-
chr2.113175261-113175491>RGPD8	22	15	17	12	39	30	50	18	0.44	0.34	-
chr2.17953901-17954051>GEN1	7	4	8	3	9	6	15	3	0.466667	-	-
chr2.182339687-182340015>ITGA4	264	123	327	51	288	107	548	183	0.481752	-	-
chr2.243168539-243168819>samemo	33	8	60	19	84	49	68	31	0.485294	-	-
**chr2.88336462-88336570>KRCC1**	**76**	**38**	**76**	**66**	**72**	**17**	**164**	**26**	**0.463415**	**0.463415**	**0,439024**
chr3.167452594-167452717>PDCD10	48	21	84	69	51	15	138	50	0.347826	-	0.369565
chr3.25637911-25639423>RARB	8	4	4	3	12	13	19	2	0.421053	0.210526	-
chr1.112991564-112991794>CTTNBP2NL	9	6	3	2	7	3	16	2	-	0.1875	0.4375
chr1.114499947-114500540>wawleybo	8	2	1	1	2	2	10	4	-	0.1	0.2
chr1.180049625-180049796>CEP350	44	29	38	7	59	29	79	7	-	0.481013	-
chr1.19470474-19470585>UBR4	103	65	85	24	139	65	178	53	-	0.477528	-
chr1.235956803-235956912>LYST	105	61	98	14	96	39	201	14	-	0.487562	0.477612
chr2.101606718-101606908>NPAS2	8	5	2	3	5	3	6	2	-	0.333333	-
chr2.231663444-231663879>CAB39	722	395	519	131	655	244	1300	161	-	0.399231	-
chr1.85127881-85128058>SSX2IP	26	2	32	16	22	19	47	15	-	-	0.468085
chr2.118864235-118864479>INSIG2	106	49	107	48	86	21	196	19	-	-	0.438776
chr2.173420100-173420447>PDK1	2	3	9	4	1	2	4	2	-	-	0.25
chr2.20756227-20757428>dawgorbu	3	3	6	1	1	2	3	2	-	-	0.333333
