# Supplementary material for: RNA-Based Biomarkers for Diagnostic Discrimination of Ischemic and Hemorrhagic Stroke: A Systematic Review
Source: J Clin Med. 2026 Feb 10;15(4):1392. doi: 10.3390/jcm15041392 (PMC12942304; doi:10.3390/jcm15041392)
Supplement: Supplementary file 1 [file jcm-15-01392-s001.zip › Table S6-New_A4_Table.pdf]

Gene Symbol	Transcript	Gene ID	HS vs. IS (FC)
**FC> 2**			**SD**
EVL	EVL-017	ENSG00000196405	8,05
RAB27A	RAB27A-002	ENSG00000069974	2,30
TBC1D8	TBC1D8-011	ENSG00000204634	2,03
**FC< (-2)**			
ANKH	ANKH-201	ENSG00000154122	-4,30
AP2B1	AP2B1-012	ENSG00000270478	-7,35
APOBEC3G	APOBEC3G-004	ENSG00000239713	-18,64
ITGB7	ITGB7-006	ENSG00000139626	-3,24
LEF1	LEF1-005	ENSG00000138795	-7,73
LEF1	LEF1-009	ENSG00000138795	-2,12
LMNB1	LMNB1-004	ENSG00000113368	-11,77
NAP1L1	NAP1L1-008	ENSG00000187109	-5,34
PTPN4	PTPN4-004	ENSG00000088179	-3,65
RHCE	RHCE-002	ENSG00000188672	-16,31
RP11-175P13.3	RP11-175P13.3-001	ENSG00000257489	-2,45
RRM1	RRM1-014	ENSG00000167325	-3,76
SLC16A10	SLC16A10-004	ENSG00000112394	-4,29
TTC39B	TTC39B-204	ENSG00000155158	-7,31
