# Supplementary material for: RNA-Based Biomarkers for Diagnostic Discrimination of Ischemic and Hemorrhagic Stroke: A Systematic Review
Source: J Clin Med. 2026 Feb 10;15(4):1392. doi: 10.3390/jcm15041392 (PMC12942304; doi:10.3390/jcm15041392)
Supplement: Supplementary file 1 [file jcm-15-01392-s001.zip › Table S7-New_A5_Table.pdf]

Gene Symbol	Transcript	Transcript ID	Transcript Biotype	Gene ID	FC	p-value
TRBV29-1	TRBV29-1-001	ENST00000566487	TR_V_gene	ENSG00000261450	-2,52	3,46E-03
TRAJ22	TRAJ22-001	ENST00000390515	TR_J_gene	ENSG00000211867	-2,50	8,23E-05
TRAC	TRAC-001	ENST00000478163	TR_C_gene	ENSG00000229164	-2,44	1,70E-05
TRAJ32	TRAJ32-001	ENST00000390505	TR_J_gene	ENSG00000211857	-2,33	2,11E-04
TRAJ35	TRAJ35-001	ENST00000390502	TR_J_gene	ENSG00000211854	-2,31	3,63E-03
TRGJP1	TRGJP1-001	ENST00000390339	TR_J_gene	ENSG00000211692	-2,29	1,40E-03
TRAJ56	TRAJ56-001	ENST00000390483	TR_J_gene	ENSG00000211835	-2,22	2,34E-03
TRAJ31	TRAJ31-001	ENST00000390506	TR_J_gene	ENSG00000211858	-2,21	1,09E-03
TRAJ45	TRAJ45-001	ENST00000390492	TR_J_gene	ENSG00000211844	-2,20	2,69E-03
TRBC2	TRBC2-001	ENST00000570020	TR_C_gene	ENSG00000260881	-2,17	6,26E-04
TRAV12-3	TRAV12-3-001	ENST00000390442	TR_V_gene	ENSG00000211794	-2,11	4,84E-04
TRAJ26	TRAJ26-001	ENST00000390511	TR_J_gene	ENSG00000211863	-2,09	2,20E-03
TRAJ36	TRAJ36-001	ENST00000390501	TR_J_gene	ENSG00000211853	-2,09	5,67E-04
TRAJ20	TRAJ20-001	ENST00000390517	TR_J_gene	ENSG00000211869	-2,08	4,15E-04
TRAJ9	TRAJ9-001	ENST00000390528	TR_J_gene	ENSG00000211880	-2,03	1,67E-04
TRAJ54	TRAJ54-001	ENST0000390484	TR_J_gene	ENSG00000211836	-2,02	2,39E-03
TRAJ3	TRAJ3-001	ENST00000390534	TR_J_gene	ENSG00000211886	-2,02	4,11E-04
TRAJ10	TRAJ10-001	ENST00000390527	TR_J_gene	ENSG00000211879	-2,00	4,94E-04
TRAJ8	TRAJ8-001	ENST00000390529	TR_J_gene	ENSG00000211881	-1,99	1,94E-03
TRAJ16	TRAJ16-001	ENST00000390521	TR_J_gene	ENSG00000211873	-1,97	3,39E-03
TRAJ53	TRAJ53-001	ENST00000390485	TR_J_gene	ENSG00000211837	-1,96	1,97E-03
TRAV14DV4	TRAV14DV4-001	ENST00000390440	TR_V_gene	ENSG00000211792	-1,96	2,22E-03
TRAJ30	TRAJ30-001	ENST00000390507	TR_J_gene	ENSG00000211859	-1,95	2,59E-04
TRAJ21	TRAJ21-001	ENST00000390516	TR_J_gene	ENSG00000211868	-1,95	1,30E-03
TRAJ1	TRAJ1-001	ENST00000390536	TR_J_gene	ENSG00000211888	-1,95	3,34E-04
TRAJ13	TRAJ13-001	ENST00000390524	TR_J_gene	ENSG00000211876	-1,93	1,68E-03
TRAJ42	TRAJ42-001	ENST00000390495	TR_J_gene	ENSG00000211847	-1,92	3,80E-03
TRAJ52	TRAJ52-001	ENST00000390486	TR_J_gene	ENSG00000211838	-1,89	1,55E-03
TRAJ38	TRAJ38-001	ENST00000390499	TR_J_gene	ENSG00000211851	-1,88	2,00E-03
TRAJ48	TRAJ48-001	ENST00000390489	TR_J_gene	ENSG00000211841	-1,87	1,50E-03
TRAJ27	TRAJ27-001	ENST00000390510	TR_J_gene	ENSG00000211862	-1,86	1,42E-03
TRAJ44	TRAJ44-001	ENST00000390493	TR_J_gene	ENSG00000211845	-1,82	3,73E-03
TRAV19	TRAV19-001	ENST00000390447	TR_V_gene	ENSG00000211799	-1,81	4,49E-03
TRAJ34	TRAJ34-001	ENST00000390503	TR_J_gene	ENSG00000211855	-1,79	3,66E-03
TRAJ18	TRAJ18-001	ENST00000390519	TR_J_gene	ENSG00000211871	-1,78	1,52E-03
TRAV13-1	TRAV13-1-001	ENST00000390436	TR_V_gene	ENSG00000211788	-1,77	4,79E-03
TRAJ7	TRAJ7-001	ENST00000390530	TR_J_gene	ENSG00000211882	-1,76	1,63E-04
TRAJ14	TRAJ14-001	ENST00000390523	TR_J_gene	ENSG00000211875	-1,74	7,33E-04

TRAV4	TRAV4-001	ENST00000390426	TR_V_gene	ENSG00000211778	-1,71	2,36E-03
TRAJ2	TRAJ2-001	ENST00000390535	TR_J_gene	ENSG00000211887	-1,71	3,01E-04
TRAJ37	TRAJ37-001	ENST00000390500	TR_J_gene	ENSG00000211852	-1,70	7,93E-04
TRBV7-8	TRBV7-8-001	ENST00000567144	TR_V_gene	ENSG00000261571	-1,70	4,14E-03
TRAJ29	TRAJ29-001	ENST00000390508	TR_J_gene	ENSG00000211860	-1,68	3,84E-03
TRAJ19	TRAJ19-001	ENST00000390518	TR_J_gene	ENSG00000211870	-1,67	4,94E-03
TRAJ24	TRAJ24-001	ENST00000390513	TR_J_gene	ENSG00000211865	-1,62	4,23E-03
TRGV7	TRGV7-001	ENST00000427089	TR_V_pseudogene	ENSG00000249978	-1,61	4,90E-03
TRAJ11	TRAJ11-001	ENST00000390526	TR_J_gene	ENSG00000211878	-1,60	2,32E-03
TRAJ17	TRAJ17-001	ENST00000390520	TR_J_gene	ENSG00000211872	-1,57	2,83E-04
TRAJ6	TRAJ6-001	ENST00000390531	TR_J_gene	ENSG00000211883	-1,50	1,26E-03
TRAJ4	TRAJ4-001	ENST00000390533	TR_J_gene	ENSG00000211885	-1,47	8,98E-04
TRAV8-3	TRAV8-3-001	ENST00000390435	TR_V_gene	ENSG00000211787	-1,46	1,35E-03
TRAJ12	TRAJ12-001	ENST00000390525	TR_J_gene	ENSG00000211877	-1,44	1,21E-03
TRAV17	TRAV17-001	ENST00000390445	TR_V_gene	ENSG00000211797	-1,36	4,83E-03
TRBV7-4	TRBV7-4-001	ENST00000566285	TR_V_gene	ENSG00000259913	-1,27	2,17E-03
TRAV2	TRAV2-001	ENST00000390424	TR_V_gene	ENSG00000211776	-1,22	1,68E-03
